# Does graduate students' satisfaction with research laboratory affect their anxiety? Findings from a cross-sectional study at a Japanese university

**DOI:** 10.3389/fpsyg.2025.1473555

**Published:** 2025-06-13

**Authors:** Li Zheng, Weida Deng

**Affiliations:** ^1^School of Education, Shanghai Normal University, Shanghai, China; ^2^Center for Teaching and Learning Development, Xiamen University, Xiamen, Fujian, China

**Keywords:** graduate student, anxiety, research laboratory, Japan, Structural Equation Modeling

## Abstract

This study investigates the relationship between graduate students' satisfaction with their research laboratories and their anxiety levels, using 2017 survey data from a Japanese university. Through correlation analysis and Structural Equation Modeling (SEM), this study examined how factors such as laboratory satisfaction, research outcome satisfaction, financial burden, and anxiety are interconnected. The findings reveal three key insights. First, graduate students report the highest levels of anxiety related to future prospects, employment, and economic conditions, and they are most likely to seek advice from parents or partners when experiencing anxiety. Second, satisfaction with the research laboratory significantly reduces anxiety, with the guidance methods of supervisors, interpersonal relationships, and research funding being the most influential factors. Satisfaction with research outcomes also plays a notable mediating role in this relationship. Third, seeking anxiety counseling is associated with increased anxiety levels, particularly when advice is sought from peers. These findings underscore the importance of the research laboratory environment in shaping graduate students' psychological wellbeing and provide a framework for understanding the mechanisms underlying anxiety development. This study highlights the need for universities to address laboratory dynamics and support systems to mitigate graduate student anxiety.

## Introduction

As a psychological and physiological state characterized by feelings of unease, apprehension, or fear, a, anxiety among college students has received increasing attention from various sectors of society in recent years which can be attributed to several reasons. First, anxiety is prevalent among the current population of college students around the world. Ramón-Arbués et al. (2020) found depression (18.4%), anxiety (23.6%), and stress (34.5%) symptoms in a sample of 1,074 Spanish university students. In a sample with 44,447 college students, the prevalence of anxiety and depression symptom was 7.7% (Wang et al., 2020). Islam et al. (2020) found in a sample with 400 first-year university students in Bangladesh that the prevalence rates of moderate to extremely severe levels of depression and anxiety were 69.5% and 61%, respectively. In Japan, a study based on 1,258 respondents from five universities showed that 42.6% of the respondents experienced depressive symptoms, and 70.7% of the respondents experienced anxiety symptoms (Kitazawa et al., 2018). Furthermore, it is imperative to highlight that multiple studies have demonstrated the prevalence of higher rates of depression, anxiety, and mental stress among graduate students compared to undergraduate students (American College Health Association, 2014; Charles et al., 2022; Evans et al., 2018). Therefore, there is significant value in addressing the anxiety levels experienced by graduate students.

Existing research has demonstrated that anxiety increases discomfort during college students' learning performance (Cohen et al., 2019; Ramón-Arbués et al., 2020; Yang et al., 2018) and negatively affects students' self-efficacy (Deer et al., 2018; Morales-Rodríguez and Pérez-Mármol, 2019). Anxiety serves as a catalyst for other detrimental behaviors that negatively affect students' physical and mental health as well, such as low social desire, alcohol use disorder, sleep problems, emotional overeating, etc. (Becker et al., 2018; Daros et al., 2019; Gao, 2021; Villarosa et al., 2014). Therefore, addressing anxiety among college students is of utmost importance for safeguarding their mental wellbeing and fostering their learning and development.

Some research has confirmed the significant impact of various types of environments, such as sports environments, learning environments, and classroom environments, on college students' anxiety (Ball and Hussey, 2020; Li et al., 2022; Yikealo et al., 2018). The learning environment significantly influences college students' academic performance and mental health, with a negative school climate and classroom environment contributing to underachievement and competitive environments increasing the risk of depression and anxiety (Barton and Bulmer, 2017; Nicita et al., 2025; Sorrenti et al., 2024; Veltri et al., 2006). Furthermore, the physical aspects of the campus, such as the presence of natural elements like rivers, lakes, and greenspaces, as well as interactions with plants, can have restorative effects on students' mental health and emotional wellbeing, particularly in the aftermath of stressful events like the COVID-19 pandemic. Therefore, it is crucial to pay attention to the influence of the environment in which graduate students are situated during their learning and living processes. Research laboratory is a place where scientific research is carried out and is usually equipped with the instruments and equipment needed to conduct experiments and observations, which is the key academic organization that play an essential role in the training of graduate students (Rodriguez et al., 2022). Thus, examining the impact of graduate students' satisfaction with research laboratory on their level of anxiety holds significant importance.

The focus of this study on Japanese graduate students is motivated by the significant role of Japanese graduate education within the Asian context. Japan established a modern higher education system modeled on western countries in the late 19th century, which was the first Asian country to establish a highly developed higher education system (Altbach and Selvaratnam, 1989; Yonezawa and Shimmi, 2016). Since the late 1980s, when the University Council initiated reforms in graduate education, the number of graduate students in Japan has increased substantially, the quality of graduate training has improved, and graduate education institutions have become more diverse (Ogawa, 1999; Suga, 2017). However, since the 1990s, Japan has experienced a slowdown in economic and social development, with social issues such as a declining birth rate and low marriage and childbirth rates becoming increasingly prominent (Date and Shimizutani, 2007; Hayashi and Prescott, 2002; Matsukura et al., 2007). For instance, from 1986 to 2015, the number of people aged 65 and above in Japan increased by 1.43 times. The proportion of newborns decreased to 23.5% in 2012. The crude marriage rate decreased from its peak of 10.5% in 1971 to 4.9% in 2017, while the crude divorce rate increased from its lowest point of 0.73% in 1963 to 1.70% in 2017. As a result, graduate students in Japan face pressure from various aspects.

When comparing Japan's graduate education system to those in Europe and the United States, several key differences emerge. Unlike the more individualistic and research-oriented approach often found in Western graduate programs, Japanese laboratories foster a collective and process-oriented environment (Arof et al., 2024; Dujarric, 2024). This environment emphasizes the development of interpersonal skills and a deep commitment to the laboratory group, which is less common in Western settings where individual achievements are often prioritized. Moreover, the integration of industry and academia in Japan's graduate education is more pronounced than in many European and American institutions (Yamaguchi and Dholakia, 2016). This integration facilitates a seamless transition from academic research to practical applications, a feature that is particularly noteworthy and contributes to Japan's reputation for innovation and technological advancement.

Amidst the economic and social development challenges faced by various Asian countries and the growing complexity of societal issues, focusing on the anxiety levels of graduate students in Japanese universities holds significant relevance for comprehending and alleviating anxiety among this demographic. This study addresses the gap in the literature, which has largely neglected the potentially heightened anxiety among graduate students and the influence of their primary learning environment, such as research laboratories, in favor of individual factors like personal traits and experiences. By investigating the correlation between Japanese graduate students' satisfaction with their research laboratory settings and their anxiety levels, this study aims to provide new insights into the environmental factors that contribute to anxiety within this population.

Building on the identified gaps in the literature regarding graduate students' anxiety and satisfaction with research laboratories, this study framed the following research questions to better understand the impact of research laboratory satisfaction on graduate students' anxiety levels and the various factors that mediate this relationship:

RQ1: What are the primary aspects of anxiety currently experienced by graduate students?
RQ2: How does graduate students' satisfaction with laboratories influence their levels of anxiety?
RQ3: What is the mechanism through which graduate students' satisfaction with laboratories affects their anxiety?

## Literature review

### The impact of anxiety on graduate student and its influencing factors

Anxiety can be divided into state anxiety and trait anxiety. State anxiety refers to a transient, intense, and unpleasant emotional state that individuals experience over a period of time. Trait anxiety, on the other hand, is a relatively stable tendency toward anxiety, characterized by individual differences and representing an emotional and psychological state that persists across different periods and situations (Spielberger, 1966). Graduate students may experience anxiety due to their specific states (academic performance, romantic relationships, interpersonal interactions, etc.) or traits (physical health, psychological wellbeing, self-efficacy, etc.) (Chi et al., 2023; Tutkun, 2019). In addition, researchers found that anxiety has a significant negative impact on graduate students' academic performance (Chapell et al., 2005; Lisnyj et al., 2020; Zagorski, 2018), and it can lead to various physical and psychological problems among graduate students, including feelings of loneliness, a decline in sleep quality, reduced life satisfaction, etc. (Bogardus et al., 2022; Gallea et al., 2021; Malik et al., 2023).

Some studies have found that graduate students' anxiety is related to their personal characteristics and experiences. For instance, some factors in graduate students' learning process, including learning pattern, expectations related to learning outcome, academic performance and academic stress have significant correlation with their level of anxiety (Ho, 2016; Öztekin, 2025; Youssef and Alibraheim, 2024). Some unhealthy lifestyles of graduate students, as embodied by irregular diet, low exercise frequency, obesity, smoking, and drinking affects their anxiety disorders and levels (Cao et al., 2025). Some studies have also found that some psychological states of graduate students, including self-esteem, psychological wellbeing, confidence, etc. can affect their anxiety (Bai et al., 2025; Sweetman et al., 2023). Furthermore, female graduate students, older graduate students, introverts, graduate students from private university, and having a mother with a high education were significantly associated with high level of anxiety (Casey et al., 2016).

### The position and role of laboratory culture in the learning environment

Laboratory culture, defined as the shared norms, values, and practices within academic research groups, plays a pivotal role in shaping graduate students' learning experiences and psychological wellbeing (Gewin, 2021; Konstantinidis, 2024; Park et al., 2017). As a micro-level learning environment, laboratories serve as the primary setting for skill development, mentorship, and academic socialization. Studies highlight those collaborative cultures emphasizing open communication and peer support correlate with higher student satisfaction and reduced anxiety (Falkner et al., 2013; Hilliard et al., 2020; Pointon-Haas et al., 2024). Conversely, hierarchical or competitive cultures may exacerbate stress by prioritizing outcomes over process, limiting autonomy, or normalizing excessive workloads (Carson et al., 2013; Chen and Liao, 2025). Thus, laboratory culture functions as both a scaffold for academic growth and a potential stressor, depending on its alignment with students' needs.

The role of laboratory culture extends to mediating institutional policies and individual outcomes. This complex interplay is evident in the way transparent communication patterns, equitable power distribution, and the alignment of research goals between supervisors and students serve as critical cultural dimensions within the laboratory setting (Nadeem, 2024; Wu et al., 2024). Additionally, laboratory culture acts as an invisible curriculum, influencing the development of not only academic competence but also the emotional resilience of students operating within high-stakes research environments (Arranz et al., 2022; González-Sanguino et al., 2021; Talevi et al., 2020). The strength of this culture can be the difference between a laboratory that produces outstanding research and one that cultivates well-rounded, emotionally resilient scientists who are prepared to tackle the complexities of the modern research landscape (Marshall et al., 2023).

### The impact of research laboratory on graduate students

The research training of graduate students (especially STEM graduate students) primarily occurs in research laboratories with laboratory groups (Borrego et al., 2017; Burt, 2017; Church, 2022). Therefore, research laboratory plays a significant role in training graduate students. Research laboratory can function as a community of practice where graduate students learn both formally and informally the nature of “good science,” while the engaging in shared research work of graduate students in the research laboratory can provide create positive learning environments for graduate students as they learn the research knowledge and skills needed to advance in their field, and was beneficial to their socialization process (Holley, 2009; Munawar et al., 2018; Rodriguez et al., 2022). And collaborative and supportive research laboratory were more likely to retain graduate students (Borrego et al., 2017; Welde and Laursen, 2008). On the contrary, graduate students reported increased indications of distress during the lockdown of research laboratory caused by COVID-19 (Suart et al., 2021).

It should be noted that the impact of the research laboratory on graduate students is not always positive. The large size of research laboratory team was likely to decrease the level of direct collaboration between teachers and graduate students, and there may be tension between collaboration and competition in research laboratories (Crede and Borrego, 2012; Louis et al., 2007; Yoo et al., 2024), which may lead to less sharing of information or resources among research laboratory group members, more individualistic approaches to work, decreased satisfaction with research laboratory group experiences (Borrego et al., 2017; Crede and Borrego, 2012). Competition in research laboratory may also lead to stratification within research laboratory groups, which means higher performing students get more opportunities to develop their skills and to share their work than graduate students who are viewed as less skilled or capable by their advisor, and may even lead to some unethical behaviors (Feldon et al., 2016; Löfström and Pyhältö, 2015).

### Research hypothesis and research framework

Based on the synthesized literature, this study proposes that graduate students' satisfaction with their research laboratory environment significantly influences their anxiety levels, mediated by the quality of laboratory culture and moderated by individual characteristics. Specifically, we hypothesize:

RH1: Graduate students exhibit higher levels of anxiety regarding economic affairs and future life planning.
RH2: Higher satisfaction among graduate students with the laboratory is associated with lower anxiety levels.
RH3: Seeking counseling from peers can reduce the anxiety level of graduate students.

**Figure 1:**
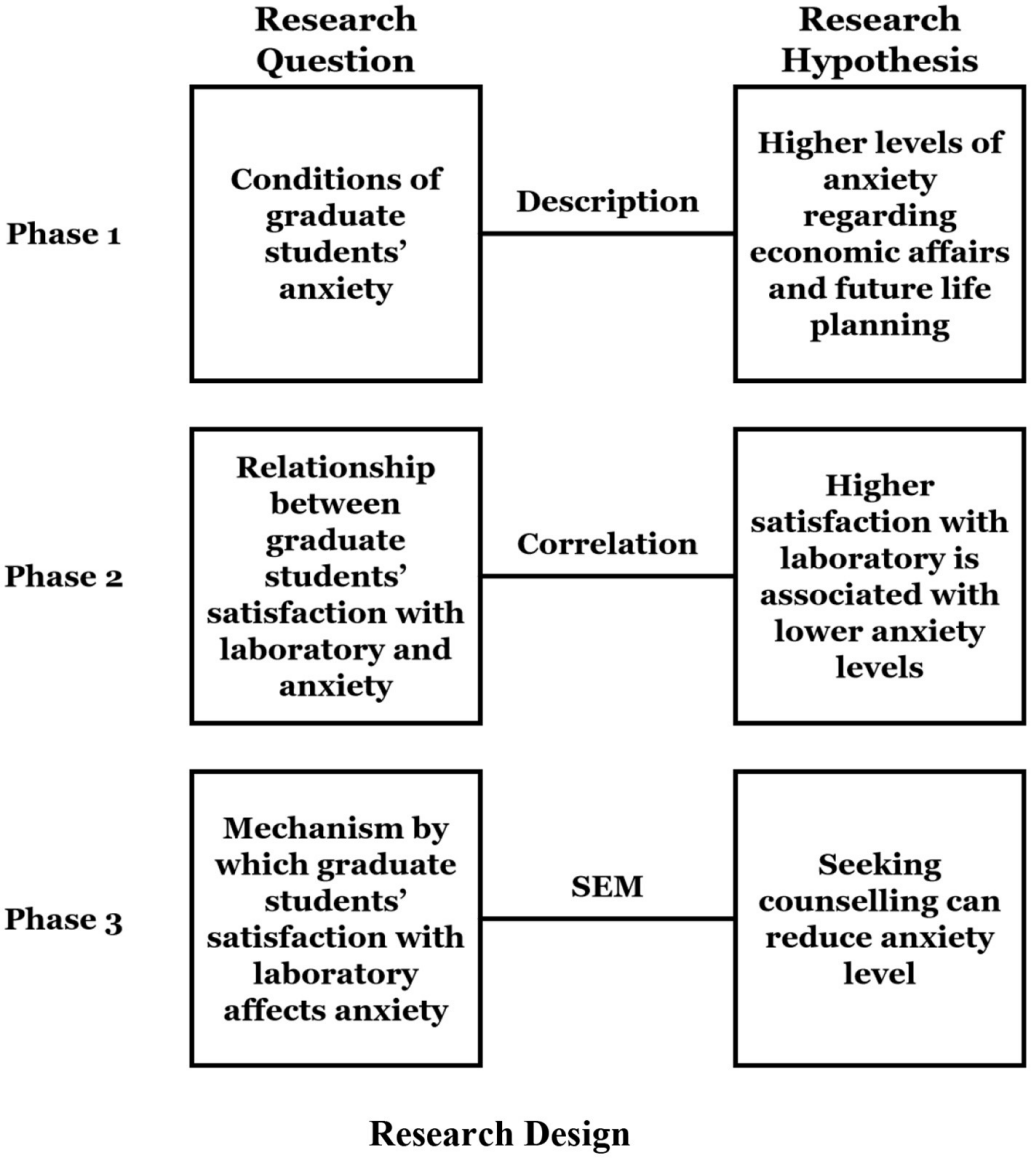
Research framework.

The research framework integrates a cross-sectional design to examine these relationships through three phases: (1) exploratory analysis of the specific conditions of graduate students' anxiety based on descriptive statistics; (2) exploration of the relationship between graduate students' satisfaction with the laboratory and their anxiety level based on correlation analysis; and (3) investigation of the mediating role of seeking counseling in the pathway through which graduate students' satisfaction with the laboratory affects their anxiety level, using Structural Equation Modeling (SEM). The research frameworks are depicted in the Figure 1.

## Research design

### Participants

In this study, we analyzed survey data gathered from a Japanese university in 2017, a year marking the continuation of an annual tradition that began in 1950. The dataset comprised 923 responses from graduate students. Table 1 provides a comprehensive overview of the sample's demographic and socioeconomic characteristics.

**Table 1:** Sample description.

**Characteristic**	**Category**	***N***	**Percentage**
Gender	Male	676	73.24
	Female	247	26.76
Education level	Master student	609	65.98
	Doctoral student	314	34.02
Major	Humanities and social science	50	5.46
	Education	25	2.73
	Law and political science	55	6.00
	Economics	16	1.75
	Integrated culture	76	8.30
	Natural science	124	13.54
	Engineering	212	23.14
	Agriculture and life science	80	8.73
	Medicine	57	6.22
	Pharmacy	31	3.38
	Mathematical science	19	2.07
	Creating science in new fields	103	11.24
	Information science	42	4.59
	Interdisciplinary information science	15	1.64
	Public policy	11	1.20
Age	21–23 years	246	26.74
	24–26 years	460	50.00
	27–29 years	120	13.04
	30–32 years	44	4.78
	33–35 years	20	2.17
	Over 36 years	30	3.26
Family address	Tokyo	272	31.26
	Kanto (except Tokyo)	310	35.63
	Hokkaido	13	1.49
	Tohoku	21	2.41
	Kinki	112	12.87
	Chugoku	102	11.7
	Shikoku	29	3.33
	Kyushu and Okinawa	11	1.26
Father's occupation	Professional and technology	226	25.08
	Education	95	10.54
	Management	292	32.41
	Affairs and administration	45	4.99
	Sales	60	6.66
	Service	43	4.77
	Security	10	1.11
	Agriculture, forestry, and fishing	6	0.67
	Production	20	2.22
	Transport	4	0.44
	Construction and mining	31	3.44
	Handling and cleaning	10	1.11
	Unemployed	59	6.55
	**Mean**	**Std. dev**.	
Family income	5,908,236	220,000	
Number of family members	3.84	8.28	

The sample of this study was predominantly male (73.24%) and consisted largely of master's students (65.98%). The most represented academic field was engineering (23.14%), with natural science (13.54%) and the creation science in new fields (11.24%) following closely. The age distribution of the sample was concentrated within the 21–29 years bracket (89.78%), reflecting the typical age range of graduate students. Geographically, the sample was heavily weighted toward students from Tokyo and the Kanto region (excluding Tokyo), accounting for 66.89% of the respondents. These areas are renowned for their economic development in Japan.

In terms of family background, the sample revealed a trend in the occupational status of the students' fathers, with management (32.41%), professional/technical (25.08%), and education (10.54%) being the most common professions. The average household size for the respondents was 3.9 members, and the mean annual income of the primary earner in the family was 5,908,236 Japanese Yen.

### Variables

#### Dependent variable

This study specifically examines the anxiety levels of graduate students, which was measured using the “level of anxiety in student life” scale (Cronbach's α = 0.831) from the survey (shown in Table 2). The scale comprises 11 items that assess the self-reported anxiety of graduate students across different domains, including academic performance, further education, employment, and interpersonal relationships.

**Table 2:** Survey items.

**Dependent variable**	**Item**	**Option**
Level of anxiety in student life	Learning (grades, credits, etc.)	1 = Extremely worried 2 = Sometimes worried 3 = Not very worried 4 = Not worried at all
	Further education	
	Employment	
	Future life	
	Interpersonal relationships with friends	
	Interpersonal relationships with faculty and staff	
	Sex, opposite sex, love, and marriage	
	Financial matters and economic independence	
	One's own personality	
	Personal health status	
	The meaning and goals of life	
**Independent variable**	**Item**	**Option**
Satisfaction with research laboratory	Research equipment	1 = Satisfied 2 = Somewhat satisfied 3 = Hard to say/Neutral 4 = Somewhat dissatisfied 5 = Dissatisfied
	Research funding	
	Interpersonal relationships	
	Tutors' guidance approach	
	Staff attitudes	
**Mediating variable**	**Item**	**Option**
Satisfaction with research outcome		1 = Satisfied 2 = Somewhat satisfied 3 = Hard to say/Neutral 4 = Somewhat dissatisfied 5 = Dissatisfied
Research financial burden	Research books purchasing expenses	
	Necessary photocopying fees and other stationery purchasing expenses	
	Expenses for surveys, experiments, etc.	
	Expenses related to academic conferences	
	Other expenses	
Consulting target after experiencing anxiety	Parents	1 = Frequently consult 2 = Sometimes consult 3 = Occasionally consult 4 = Never consult
	Siblings	
	Collaborative partners and spouse	
	Student counseling services	
	Faculty and staff	
	Friends in the same discipline or research laboratory	
	Friends from student organizations	
	Friends outside of university	
	Seniors	
	Romantic partner	

#### Independent variable

In this study, the satisfaction of graduate students with their research laboratories is considered as the core explanatory variable, which was measured in the survey using the “satisfaction with research laboratory” scale (shown in Table 2) (Cronbach's α = 0.739). The scale consists of five items that assess the extent of graduate students' self-reported satisfaction with various aspects of the research laboratory, including research equipment, research funding, interpersonal relationships, the tutors' guidance approach, and staff attitudes.

#### Mediating variable

Several studies have demonstrated the relationship between satisfaction with research outcomes, research time, research financial burden, and anxiety, as well as the correlation between these variables (Feizi and Elgar, 2023; Mushtaq, 2023; Ou et al., 2024; Wang and Wang, 2018). Thus, this study incorporates three mediating variables, namely “satisfaction with research outcomes,” “research time,” and “research financial burden” to explore their potential impact on graduate students' anxiety (shown in Table 2). These variables were assessed based on self-reported data provided by the respondents, ensuring their subjective perspectives were considered. To measure “satisfaction with research outcomes,” the item “satisfaction with research outcomes” was included. As a proxy variable for “research time,” the study utilized “average weekly research time.” To capture the various financial burdens of graduate students, including expenses related to purchasing research-required books, photocopying or obtaining necessary stationery materials, as well as costs associated with surveys, experiments, and other research activities, the “annual personal financial burden” was employed. In addition, to investigate the coping strategies of anxiety employed by graduate students, this study includes the proxy variable of seeking counseling from various sources (such as parents, siblings, teachers, etc.) as a measure of coping mechanisms (shown in Table 2), which is assessed using the “consulting target after experiencing anxiety.”

### Data analysis

This study commences by presenting the anxiety levels of graduate students and their inclination to seek consultation from others, utilizing descriptive statistics and correlation analysis. Subsequently, Structural Equation Modeling (SEM) is employed to explore the mediating effects of graduate students' satisfaction with research outcomes, research time, and research financial burden. The analysis of the SEM relies on establishing a satisfactory fit of the constructed model. Three types of fit indices, namely absolute fit indices, incremental fit indices, and parsimonious fit indices, are primarily utilized to assess the adequacy of the SEM model. In this study, the following fit indices were chosen for each category: Root Mean Square Error of Approximation (RMSEA), Normed Fit Index (NFI), and Parsimonious Comparative Fit Index (PCFI). The results of the model indicate that the RMSEA, NFI, and PCFI values are 0.078, 0.625, and 0.562, respectively, all falling within an acceptable range, which suggest that the model in this study exhibits a good fit, enabling further analysis to be conducted.

## Findings

### Anxiety of graduate students and their coping measures

Table 3 presents the descriptive statistics of anxiety among graduate students. The results indicate that within the study sample, the respondents exhibit higher levels of anxiety in “future life,” “employment,” and “financial matters and economic independence.” Specifically, the proportions of respondents reporting being “extremely worried” about these three aspects are 46.7%, 38.5%, and 31.0%, respectively. On the other hand, the respondents displayed the lower levels of anxiety in “further education,” “interpersonal relationships with friends,” and “interpersonal relationships with faculty and staff.” In these areas, the proportions of respondents reporting being “not worried at all” are 34.6%, 26.6%, and 22.8%, respectively. The results of the descriptive statistics indicate that graduate students generally experienced lower levels of anxiety in relation to their current studies and interpersonal relationships, confirming the RH 1 of this study. However, they demonstrated higher levels of anxiety when it comes to future prospects, employment, and related concerns.

**Table 3:** Anxiety status of respondents.

**Item**	**Anxiety status**			
	**Extremely worried**	**Sometimes worried**	**Not very worried**	**Not worried at all**
Learning (grades, credits, etc.)	23.8%	27.20%	27.70%	21.30%
Further education	15.8%	21.20%	28.40%	34.60%
Employment	38.5%	34.20%	17.20%	10.10%
Future life	46.7%	35.70%	11.80%	5.70%
Interpersonal relationships with friends	7.0%	20.50%	46.00%	26.60%
Interpersonal relationships with faculty and staff	11.1%	25.20%	40.90%	22.80%
Sex, opposite sex, love, and marriage	17.9%	39.50%	26.50%	16.10%
Financial matters and economic independence	31.0%	38.80%	20.40%	9.90%
One's own personality	18.7%	29.00%	33.80%	18.50%
Personal health status	13.6%	31.60%	36.40%	18.40%
The meaning and goals of life	22.2%	33.30%	28.60%	15.90%

Table 4 presents the results of descriptive statistics of the respondents consulting others after experiencing anxiety. The results indicate that parents, lovers, and spouses were the primary sources of consultation for graduate students after experiencing anxiety, and the proportion of respondents who chose “frequent consult” was 18.1%, 13.6%, and 11.8%, respectively. It is noteworthy that graduate students had a relatively lower tendency to seek consultation from the “student counseling services” or “faculty and staff” after experiencing anxiety, with only 1.8% of students reporting “frequently consult” with them. This suggests that graduate students were more inclined to seek advice from family and friends rather than specialized counseling services or faculty members at their institution after experiencing anxiety.

**Table 4:** Consulting target after experiencing anxiety.

**Consulting target**	**Frequently consult**	**Sometimes consult**	**Occasionally consult**	**Never consult**
Parents	18.1%	21.2%	34.3%	26.4%
Siblings	3.3%	9.3%	17.7%	69.6%
Collaborative partners and spouse	11.8%	12.7%	9.4%	66.2%
Student counseling services	1.8%	3.6%	10.0%	84.6%
Faculty and staff	1.8%	9.1%	33.4%	55.7%
Friends in the same discipline or research laboratory	11.5%	29.2%	36.6%	22.8%
Friends from student organizations	6.5%	17.1%	19.0%	57.4%
Friends outside of university	11.7%	26.4%	32.2%	28.7%
Seniors	6.0%	21.5%	30.2%	42.3%
Romantic partner	13.6%	14.3%	13.0%	58.1%

This study explored the relationship between anxiety and satisfaction with research laboratory among graduate students using correlation analysis. The results obtained (shown in Table 5) indicate significant negative correlations between anxiety and both satisfaction with the research laboratory (coefficient = −0.273, *p* < 0.05) and the research result (coefficient = −0.308, *p* < 0.05). Specifically, higher satisfaction with the research laboratory is associated with lower levels of anxiety and higher satisfaction with the research outcome. In addition, the results indicate significant negative correlations between the research financial burden and graduate students' satisfaction with the research laboratory (coefficient = −0.353, *p* < 0.05). Specifically, a higher perception of financial burden in research is associated with lower satisfaction with the research laboratory. Furthermore, graduate students' satisfaction with the research laboratory exhibited a significant positive correlation with their satisfaction with the research outcome (coefficient = 0.234, *p* < 0.05), suggesting that greater satisfaction with the research laboratory is linked to higher satisfaction with the research outcome. The findings confirm the RH 2 of this study.

**Table 5:** Results of correlation analysis.

**Variable**	**Anxiety**	**Research time**	**Research financial burden**	**Satisfaction with research laboratory**	**Consulting after experiencing anxiety**
Research time	−0.011				
Research financial burden	0.023	0.064			
Satisfaction with research laboratory	−0.273^**^	0.028	−0.353^**^		
Consulting after experiencing anxiety	0.082^*^	−0.022	0.010	0.000	
Satisfaction with research outcome	−0.308^**^	0.101^*^	0.016	0.234^***^	0.076

^*^p <0.1; ^**^p <0.05; ^***^p <0.01.

### Influence mechanism of research laboratory on graduate students' anxiety

Table 6 displays the findings of SEM, which elucidates the relationships between graduate students' satisfaction with the research laboratory and various aspects of their research experience. The model indicates that satisfaction with the research laboratory significantly impacts the research burden, the level of satisfaction with research outcomes, and the average number of weekly research hours. Specifically, a higher level of satisfaction with the research laboratory is associated with a reduced perception of research burden (β = −0.478, *p* < 0.05) and an increased satisfaction with research outcomes (β = 1.093, *p* < 0.05). Furthermore, the results reveal that graduate students' anxiety is influenced by their consulting behaviors, satisfaction with research outcomes, and the research laboratory environment. Consulting positively correlates with anxiety levels (β = 0.288, *p* < 0.05), whereas satisfaction with both research outcomes (β = −0.122, *p* < 0.05) and the research laboratory (β = −0.401, *p* < 0.05) are inversely related to anxiety.

**Table 6:** Results of SEM.

**To**	**Path**	**From**	**Coefficient**	**Standard error**	**Critical ratio**	***p***
Research financial burden	←	Satisfaction with research laboratory	−0.478	0.134	−3.567	^***^
Satisfaction with research outcome	←		1.093	0.172	6.364	^***^
Research time	←		−0.03	0.094	−0.319	0.750
Consulting target after experiencing anxiety	←	Research financial burden	0.005	0.02	0.240	0.810
	←	Satisfaction with research outcome	0.026	0.014	1.873	0.061
	←	Research time	−0.003	0.015	−0.206	0.836
Anxiety	←	Consulting target after experiencing anxiety	0.288	0.068	4.225	^***^
	←	Research financial burden	−0.022	0.027	−0.833	0.405
	←	Research time	−0.009	0.019	−0.487	0.627
	←	Satisfaction with research outcome	−0.122	0.021	−5.923	^***^
	←	Satisfaction with research laboratory	−0.401	0.075	−5.319	^***^
Staff attitudes	←	Satisfaction with research laboratory	1.000			
Tutors' guidance approach	←		2.086	0.232	9.005	^***^
Interpersonal relationships	←		1.865	0.209	8.927	^***^
Research funding	←		1.439	0.19	7.564	^***^
Research equipment	←		1.263	0.165	7.655	^***^
Parents	←	Consulting target after experiencing anxiety	1.000			
Siblings	←		0.621	0.106	5.859	^***^
Collaborative partners and spouse	←		0.816	0.143	5.692	^***^
Student counseling services	←		0.231	0.068	3.39	^***^
Faculty and staff	←		0.787	0.111	7.058	^***^
Friends in the same discipline or research laboratory	←		1.766	0.206	8.575	^***^
Friends from student organizations	←		1.394	0.174	7.995	^***^
Friends outside of university	←		1.382	0.175	7.900	^***^
Seniors	←		1.562	0.187	8.364	^***^
Romantic partner	←		1.221	0.173	7.075	^***^

^***^p <0.001.

The model also highlights the relative importance of specific indicators within the latent variables of consulting and satisfaction with the research laboratory. For the research laboratory, the following factors were found to be particularly influential: “tutors' guidance approach” (β = 2.086, *p* < 0.05), “interpersonal relationships” (β = 1.865, *p* < 0.05), and “research funding” (β = 1.439, *p* < 0.05). In terms of consulting sources, “friends in the same discipline or laboratory” (β = 1.766, *p* < 0.05), “seniors” (β = 1.562, *p* < 0.05), and “friends from student organizations” (β = 1.394, *p* < 0.05) emerged as the most significant contributors. The findings reject the RH 3 of this study, and underscore the nuanced ways in which the research environment and interpersonal dynamics can affect graduate students' mental health and research experience.

### Summary

Table 7 presents a summary of the direct, indirect, and total effects of graduate students' satisfaction with research laboratory on their anxiety. Graduate students' satisfaction with research laboratory was found to have a significant negative direct effect on anxiety (β = −0.401, *p* < 0.05), indicating that higher satisfaction levels among graduate students in their research laboratory were associated with a significant reduction in anxiety. In addition, graduate students' satisfaction with research laboratory demonstrated a negative but not significant indirect effect on anxiety through research burden (β = −0.138, *p* > 0.05), a significant negative effect through satisfaction with research outcomes (β = −0.133, *p* < 0.05), and a weak positive but not significant indirect effect through average weekly research time (β = 0.000, *p* > 0.05). Lastly, the total effect of graduate students' satisfaction with research laboratory on anxiety was negative, suggesting that higher levels of graduate students' satisfaction with research laboratories were associated with lower levels of anxiety.

**Table 7:** Summary of SEM results.

**Effect**	**Coefficient**	**Significant**
Direct effect	−0.401	√
Indirect effect (research financial burden)	−0.138	×
Indirect effect (research time)	0.000	×
Indirect effect (satisfaction with research outcome)	−0.133	√
Indirect effect (consulting target after experiencing anxiety)	0.008	×
Total effect	−0.665	

## Discussion

The characteristics of anxiety among graduate students and their choice of consultation sources after experiencing anxiety are believed to be influenced by the economic and social background that Japanese university students face, as well as the primary causes of anxiety. Researchers have pointed out that anxiety among university students is not only influenced by genetic and family environmental factors but also by the socio-cultural environment (Twenge, 2000). Japanese university students are currently facing a series of pressures related to employment, childbirth, housing, and retirement, leading to relatively higher levels of anxiety regarding their future prospects, employment, and financial conditions. Moreover, students share common experiences with family members, spouses, and parents in these areas, and these individuals can provide social support as coping resources (Herman-Stahl and Petersen, 1996), which lead them to choose them as consultation sources for anxiety, seeking social support to alleviate their anxiety.

The negative correlation observed between graduate students' satisfaction with their research laboratory and anxiety might stem from the fact that practical laboratory experiences strengthen specific academic skills. This acquisition of skills, especially specialized know-how, can foster greater self-confidence and strengthen self-efficacy (Akinwumi and Ibrahim, 2024; Meechan et al., 2011). Such enhanced confidence and capabilities may directly reduce students' anxieties concerning key areas identified by the scale—employability, future life, economic independence, and life goals—thereby mitigating related anxiety. Laboratory practices serve as contexts for specialization and training in specific skills, thereby increasing students' professional potential, enabling them to acquire specialized know-how, which could lead to better employment opportunities and the desired economic independence (Bugaj and Nikendei, 2016; Greco and Reasoner, 2010; Herrmann-Werner et al., 2013). Furthermore, research laboratories foster action and the development of practical skills, positively impacting the consolidation of knowledge.

This study identified both the direct effect of graduate students' satisfaction with research laboratory on anxiety and the indirect effect mediated by satisfaction with research outcome. The higher the satisfaction levels of graduate students with their research laboratories and research outcomes, the lower their anxiety. This study considers graduate students' satisfaction with research outcome as a mechanism that reduces anxiety by enhancing students' achievement motivation. Achievement motivation refers to an individual's drive to pursue expected goals and serves as an intrinsic driving force for goal attainment (Song et al., 2015). Specifically, it manifests as a need for achievement, characterized by a strong desire for competence and success in goal-oriented tasks, while exhibiting a high aversion to risk and failure (Janman, 1987). Scientific research constitutes a significant aspect of graduate students' lives, and research outcomes represent the primary achievements they aspire to obtain. Previous research has demonstrated the inhibitory effect of achievement motivation on anxiety among graduate students (Khalaila, 2015). This study proposes that satisfaction with research outcome mediates the relationship between graduate students' satisfaction with research laboratory and anxiety by enhancing their achievement motivation, ultimately leading to a reduction in anxiety levels.

This study further revealed that seeking anxiety-related consultations increases anxiety among graduate students, with the three most influential categories of consultation sources being “friends in the same discipline or research laboratory,” “seniors,” and “friends from student organizations,” all of which fall under the category of “peers.” This finding suggests that consulting peers may inadvertently amplify individual anxiety, potentially due to the phenomenon known as “co-rumination.” Co-rumination refers to an unhealthy social support process in which individuals repeatedly discuss their stressors or problems with others, but both parties become fixated on the problems and emotions themselves rather than seeking solutions, thereby reinforcing negative experiences (Davidson et al., 2014; Waller et al., 2014). For instance, when women repeatedly share negative self-perceptions and negative emotions about their appearance and weight with a friend who actively engages in the discussion, both individuals become deeply immersed in the negative experience, leading to increased levels of depression and anxiety (Rudiger and Winstead, 2013). Graduate students share a wide range of common experiences with their peers in academics, daily life, and other aspects, and they face similar challenges such as academic work, research, and daily life. As a result, they are more likely to engage in conversations about the same issues, which can lead to the development of similar negative emotions and ultimately increase anxiety levels.

The finding that seeking help from institutionalized spaces, such as student counseling services, was not significantly associated with reduced anxiety among graduate students warrants careful discussion. This result might suggest that while these services are available, they may not be the primary or most effective resource for anxiety management for this specific population or context (Cronin et al., 2021; El-Hachem et al., 2023). Reasons for this could be that students are less aware of these services and perceive barriers to accessing them. It could also indicate that the nature of graduate student anxiety is complex and may require more tailored interventions than general counseling services typically offer. This highlights a potential gap in the effectiveness or utilization of existing institutional support structures for anxiety management in this setting (Cohen et al., 2022).

In line with the Affective Commitment Theory and Job Satisfaction Theory, an individual's contentment with their work environment can foster affective commitment, which subsequently impacts their performance and perseverance (Meyer and Allen, 1997). Job satisfaction is shaped by a multitude of factors, encompassing the work environment, interpersonal relationships, and perceived support (Spector, 1997). Moreover, an organization's resources, such as the quality of its equipment and funding, are pivotal to its competitive edge (Wernerfelt, 1984). This rationale underpins the decision to identify satisfaction with the research laboratory as the independent variable, while treating the research financial burden and research time as mediating variables. Acknowledging the legitimacy of alternative theoretical frameworks, this study is crafted to explore the function of graduate student satisfaction as a central explanatory factor. This study contends that this perspective offers profound insights into the elements that enhance a productive and satisfying research environment for graduate students.

This study indicated that the indirect effect of the research burden on anxiety was negative; it was not statistically significant, suggesting that the financial and time burdens of research do not play a strong role in mediating anxiety. Additionally, satisfaction with research outcomes emerged as a significant negative predictor of anxiety, indicating that when students are pleased with their research results, their anxiety levels decrease (Kaur et al., 2023). Interestingly, the average weekly research time had a weak positive indirect effect on anxiety, which, although not significant, hints at the potential for excessive research time to contribute to anxiety (Cooper et al., 2023). These results underscore the importance of fostering a research environment that promotes satisfaction with outcomes to mitigate anxiety among graduate students.

This study resonates with foundational psychological theories that emphasize environmental influences on mental health. The observed inverse relationship between laboratory satisfaction and anxiety aligns with Social Support Theory (Cobb, 1976), wherein institutional support systems buffer psychological distress. Specifically, supervisors' guidance methods and cohesive peer relationships mirror the theory's emphasis on instrumental and emotional support, respectively. Furthermore, the mediating role of research outcome satisfaction echoes Self-Determination Theory (Deci and Ryan, 1985): when students perceive progress in autonomy (via flexible guidance) and competence (via funded research opportunities), intrinsic motivation is enhanced, thereby reducing anxiety rooted in uncertainty. Paradoxically, the association between seeking peer advice and heightened anxiety may reflect Stress-Coping Theory (Lazarus and Folkman, 1984): in cultures prioritizing collective harmony (e.g., Japan), students might avoid disclosing vulnerabilities to peers to maintain social equilibrium, inadvertently amplifying stress through unresolved rumination.

## Conclusion

This study utilized descriptive statistics, correlation analysis, and Structural Equation Modeling (SEM) to explore anxiety levels, coping strategies, and the impact of graduate students' satisfaction with their research laboratories on their anxiety in a Japanese research university, using data from a Japanese university in 2017. The findings revealed several key insights. First, graduate students exhibited the highest levels of anxiety in their future prospects, employment, and financial conditions. Furthermore, when experiencing anxiety, they were more inclined to seek support from family and spouses rather than teachers or school consulting services. Secondly, graduate students' satisfaction with research laboratory had a significant negative impact on their anxiety, while satisfaction with research outcome plays a significant mediating role. Additionally, consulting was found to significantly increase graduate students' anxiety. Lastly, within the specific facets of graduate students' satisfaction with research laboratory, “tutors' guidance approach,” “interpersonal relationships,” and “research funding” were identified as having relatively higher weights, while among the sources of consulting, “friends in the same discipline or laboratory,” “seniors,” and “friends from student organizations” carried relatively higher weights.

This study contributes to the literature in three novel dimensions. First, it pioneers the integration of laboratory satisfaction as a systemic environmental factor into anxiety research among graduate students, whereas previous studies predominantly focused on individual psychological traits or general academic stressors. Secondly, the paradoxical finding that formal consulting services exacerbate anxiety challenges conventional assumptions about counseling efficacy in academic settings, prompting a re-examination of cultural barriers and implementation quality in Japanese higher education contexts. Thirdly, methodologically, we advance the operationalization of graduate anxiety by employing SEM to disentangle the mediating mechanism between laboratory satisfaction dimensions and anxiety manifestations, enabling a structural understanding beyond linear correlations. These innovations collectively shift the discourse from individual-centric to institution-environment interplay perspectives, offering actionable pathways for universities to redesign support systems through evidence-based environmental modifications rather than solely relying on psychological interventions.

## Limitation and future research

There are still some limitations of this study. First, given that the data primarily relies on a questionnaire survey and respondents' self-assessments, it is important to acknowledge the possibility of self-reporting bias or deviation. And it is worth noting that the data was collected in 2017 and may not necessarily reflect the most current circumstances. Additionally, a significant gender ratio difference in the sample could influence the research results, potentially leading to biased findings. This bias may affect the generalizability of the study's conclusions. Furthermore, the uneven distribution of disciplines among the participants could impact the research results, introducing discipline-specific biases that affect the interpretation and generalizability of the findings. As for the discipline distribution across Japanese laboratories, with a significant presence of Engineering and a varied representation of other fields, including Humanities and Social Sciences, Natural Sciences, and Information Science, may influence the sampling process and the broader implications of our study. This imbalance could affect the generalizability of our findings, highlighting the need for a more equitable representation across disciplines in future research to ensure comprehensive insights and informed policy-making.

Secondly, graduate students' satisfaction with research laboratory encompasses various dimensions. However, due to data limitations, this study focused solely on exploring graduate students' satisfaction with research outcome, research financial burden, and research time, without considering their satisfaction with other aspects of research laboratory. Thirdly, this study is limited by its use of a sample exclusively from single Japanese university, which may result in potential biases in the research findings. Future studies could benefit from implementing more rigorous research designs, as well as utilizing more representative samples to further investigate the relationship between graduate students' satisfaction with research laboratory. This approach would contribute to refining the framework of influencing factors that contribute to graduate students' anxiety.

Another limitation of this study pertains to the description of the specific laboratory practices integrated within the study programs of the chosen degree courses. While the focus was on the perceived outcomes (satisfaction and anxiety) related to these practices, the study did not provide a detailed characterization of the practices themselves across the different disciplines and institutions in Japan. Laboratory practices can vary significantly between fields such as engineering and the social sciences, and even within disciplines, specifics like common elements, allocated credits, working hours, and assessment weight can differ. Future research could benefit from a more detailed examination of the specific characteristics of laboratory practices, potentially correlating variations in structure, intensity, and assessment methods with student mental status to provide deeper insights into the mechanisms at play.

## Data Availability

Publicly available datasets were analyzed in this study. This data can be found here: https://ssjda.iss.u-tokyo.ac.jp/Direct/gaiyo.php?eid=1310&lang=eng.
